# Validation of Walking Trails for the Urban Training^TM^ of Chronic Obstructive Pulmonary Disease Patients

**DOI:** 10.1371/journal.pone.0146705

**Published:** 2016-01-14

**Authors:** Ane Arbillaga-Etxarri, Jaume Torrent-Pallicer, Elena Gimeno-Santos, Anael Barberan-Garcia, Anna Delgado, Eva Balcells, Diego A. Rodríguez, Jordi Vilaró, Pere Vall-Casas, Alfredo Irurtia, Robert Rodriguez-Roisin, Judith Garcia-Aymerich, Urban Training™ Study Group

**Affiliations:** 1 Centre for Research in Environmental Epidemiology (CREAL), Barcelona, Spain; 2 Pompeu Fabra University (UPF), Barcelona, Spain; 3 CIBER Epidemiology and Public Health (CIBERESP), Barcelona, Spain; 4 FCS Blanquerna, Research Group in Physical Activity and Health (SAFE), Ramon Llull University, Barcelona, Spain; 5 Pneumology department, Hospital Clínic of Barcelona, Barcelona, Spain; 6 August Pi i Sunyer Biomedicine Research Institution (IDIBAPS), University of Barcelona (UB), Barcelona, Spain; 7 CIBER Respiratory diseases (CIBERES), Bunyola, Illes Balears, Spain; 8 IMIM (Hospital del Mar Medical Research Institute), Barcelona, Spain; 9 Pneumology department, Hospital del Mar, Barcelona, Spain; 10 International University of Catalunya (UIC), Barcelona, Spain; 11 Catalan National Institute of Physical Education (INEFC), Barcelona, Spain; Clinica Universidad de Navarra, SPAIN

## Abstract

**Purpose:**

Accessible interventions to train patients with chronic obstructive pulmonary disease (COPD) are needed. We designed urban trails of different intensities (low, moderate and high) in different types of public spaces (boulevard, beach and park). We aimed to validate the trails’ design by assessing the physiological response to unsupervised walking trails of: (1) different intensities in COPD patients, and (2) same intensity from different public spaces in healthy adults.

**Methods:**

On different days and under standardized conditions, 10 COPD patients walked the three intensity trails designed in a boulevard space, and 10 healthy subjects walked the three intensity trails in three different spaces. We measured physiological response and energy expenditure using a gas analyzer. We compared outcomes across trails intensity and/or spaces using mixed-effects linear regression.

**Results:**

In COPD patients, physiological response and energy expenditure increased significantly according to the trails intensity: mean (SD) peak $\dot{\text{V}}$O_2_ 15.9 (3.5), 17.4 (4.7), and 17.7 (4.4) mL/min/kg (p-trend = 0.02), and MET-min 60 (23), 64 (26), 72 (31) (p-trend<0.01) in low, moderate and high intensity trails, respectively. In healthy subjects there were no differences in physiological response to walking trails of the same intensity across different spaces.

**Conclusions:**

We validated the trails design for the training of COPD patients by showing that the physiological response to and energy expenditure on unsupervised walking these trails increased according to the predefined trails’ intensity and did not change across trails of the same intensity in different public space. Walkable public spaces allow the design of trails that could be used for the training of COPD patients in the community.

## Introduction

Chronic obstructive pulmonary disease (COPD) is a significant cause of morbidity and mortality worldwide [1,2]. The regular practice of physical activity improves COPD prognosis by reducing the risk of COPD exacerbations and mortality [3]. Hence, physical activity constitutes an important outcome in COPD management. In contrast, research about interventions designed to increase physical activity in COPD patients is relatively still scarce, of poor quality and/or shows inconsistent results [3].

Recent findings support regular walking as a training modality to increase physical activity in COPD patients. Ground-based indoor, Nordic and standard supervised walking trainings have been shown to result in physiological benefits [4–6]. Importantly, walking is an extended practice well integrated into the daily life routine of the elderly in many countries [7–10]. Therefore, interventions promoting walking in public spaces are accessible to a wider range of patients than hospital-based programs [11] and are more conducive to compliance because they easily fit into patients’ daily life.

To our knowledge, only two studies have assessed walking in public spaces to train COPD patients. Breyer *et al*. reported an improvement in exercise capacity in COPD patients after a supervised intervention of Nordic Walking [12]. Pleguezuelos *et al*. observed that COPD patients who had received information about urban walking circuits after a rehabilitation program improved exercise capacity as compared to those who were not informed [13]. However, it is not yet known whether unsupervised walking can induce a training effect.

We hypothesized that urban walkable public spaces allow the design of trails that could be used for the training of COPD patients in unsupervised conditions. As part of the Urban Training™ on-going clinical trial (NCT01897298) we designed urban trails of different intensities (low, moderate and high) for the training of COPD patients in different types of walkable public spaces (boulevard, beach and park). The present study aimed to validate the design of trails by assessing the physiological response to and the energy expenditure on unsupervised walking trails of different intensities in COPD patients. As a secondary objective, we also assessed the physiological response to walking trails of the same intensity designed in different spaces in young healthy subjects.

## Methods

### Population

We recruited 10 clinically stable patients with a diagnosis of COPD according to the American Thoracic Society and European Respiratory Society (ATS/ERS) criteria [14] from the outpatient clinics of Hospital del Mar, Barcelona. Exclusion criteria were: musculoskeletal limitation for walking, use of long-term oxygen therapy and intolerance of the facemask. For the secondary objective, we recruited 10 healthy subjects from the Centre for Research in Environmental Epidemiology (CREAL), Barcelona. We excluded subjects with a diagnosis of any chronic condition that could affect exercise capacity and/or heart rate. The study and the use of oral consent were approved by the Clinical Research Ethical Committee of Parc de Salut Mar, and oral informed consent was obtained from all subjects. The individual in S2 Image has given written informed consent to publish these case details.

### Design of trails

A multidisciplinary team formed by respiratory physiotherapists, pulmonologists, family physicians, sport science experts, epidemiologists and urban planners identified public spaces close to the recruitment centers potentially suitable for designing walking trails. Following the World Health Organization and the Healthy Cities movement recommendations [15,16] we excluded spaces with at least one of the following environmental restrictions that might negatively influence walking [15–21]: (i) no accessibility by public transportation; (ii) lack of vegetation; (iii) absence of benches or fountains; (iv) presence of very long or high slopes; (v) paucity of elements that facilitate exercise intensity (stairs, ramps or sand); (vi) high levels of pollution, noise or car traffic; and, (vii) safety or criminality issues. Ultimately, we selected three types of walkable public spaces: boulevard, beach and park.

In each space, we measured all urban elements of intensity (stairs, ramps and types of surfacing) in terms of length, slope and height using a laser rangefinder (Bosch PLR 50, Robert Bosch GmbH, Germany). In parallel, we developed an *ad hoc* scoring table to classify the intensity of each urban element based on the energy expenditure requirements for the physical activities that each urban element demanded, namely: walking on level ground, stair/slope climbing and descending and walking on sand [22].

Finally, we designed 3 intensity trails (low, moderate and high) at each public space, combining the urban elements previously scored. In order to facilitate access and achieve a training effect, we designed all trails as continuous and circular, with several points of access and with no traffic lights or other obstacles to walking (see S1 Text, which includes more details on length, content and depiction of the trails; see S1 Table, describing characteristics of the three intensity trails of each three public spaces; see S1 Image, reproducing the trails from a boulevard, beach and park space).

### Study design and Procedures

The study was structured over four different days with at least one resting day in-between. On Day 1, COPD patients performed a 6-min walking test (6MWT). On Days 2 to 4, they walked on the low, moderate and high intensity trails from a boulevard space (total of 3 trails) in random order, using random numbers generated with Stata 9.1 (StataCorp, College Station, TX, USA). Patients received trails maps and were informed about the position, frequency and characteristics of intensity elements and were instructed by the technicians to maintain a constant self-regulated walking speed during the trail. Before starting, they stood up for 2 min at the starting point. During the walk, two members of the research team walked 2 m behind the patients without interfering in their pace. If patients needed to stop for resting, they could sit on a portable chair carried by technicians. The walking period ended when patients reached again the starting point or decided to stop walking due to symptoms. Thereafter, they recovered silently while sitting for 5 min (see S2 Image showing a COPD patient walking on level, climbing stairs and resting in a trail).

To avoid overloading the COPD patients, young healthy subjects were asked to walk on the three intensity trails (low, moderate and high) of the three types of walkable public spaces (boulevard, beach and park; total of 9 trails) to answer the secondary objective. Instructions and procedures were standardized as for the COPD patients.

### Measurements

We collected the following information: sociodemographic and clinical data by using questionnaires and medical records, weight and height by physical examination, dyspnea by the modified Medical Research Council scale (mMRC, 0 to 4), lung function by forced spirometry with bronchodilator response, and exercise capacity by the 6MWT following standards [23]. In COPD patients, during the 6MWT and the walks on the trails, a portable gas analyzer (MetaMax® 3B, CORTEX, Germany) with a facemask, adjusted to patient’s face size, collected breath-by-breath oxygen uptake per weight ($\dot{\text{V}}$O_2_), carbon dioxide production ($\dot{\text{V}}$CO_2_), ventilation ($\dot{\text{V}}$E) and the energy expenditure measured by Metabolic Equivalent Tasks (METs). Both in COPD patients and healthy subjects, heart rate (HR) during walks was obtained from a Polar Belt (Polar RS800CX, Polar Electro, Finland). We measured dyspnea and lower limb fatigue using modified Borg scale [24] immediately after 6MWT and each walk. We also measured the steps during trails using an electronic step counter (Walking style X Omron, Japan). Data collection followed strict quality control procedures, including calibration of the gas analyzer before each test and check of on-line telemetry data recorded by the gas analyzer.

### Statistical Analysis

Sample size was estimated with the program GRANMO7.10 [25] using $\dot{\text{V}}$O_2_ as the primary outcome measure. Using a standard deviation of $\dot{\text{V}}$O_2_ in COPD patients of 3 mL/min/kg [26], accepting an alpha risk of 0.05 and a beta risk of 0.2 in a two-sided test for repeated measures, a total of 5 subjects would be sufficient to detect a statistically significant difference in $\dot{\text{V}}$O_2_ between trails higher than or equal to 4 mL/min/kg, which has previously been reported between activities of low and moderate intensity, and between moderate and high intensity activities [26]. The calculated figure was increased up to 10 patients. For the secondary objective with healthy subjects, peak HR was used as the primary outcome measure. Using data from the literature [27] and the same statistical assumptions, a total of 8 subjects were required, which was rounded up to 10.

The subjects’ characteristics and physiological response to walks and 6MWT are presented as frequencies and percentages for qualitative variables, mean and SD for normal distributed quantitative variables and median and 25th-75th percentiles for non-normal distributed quantitative variables. We compared variables of physiological response across trails intensity and/or space using mixed-effects linear regression models, accounting for repeated observations within subject. In COPD patients, the primary outcomes were the peak of relative $\dot{\text{V}}$O_2_, $\dot{\text{V}}$E, HR, and METs-min. Secondary outcomes included time walking, time for breaks, walking speed, and total number of steps during the walk, as well as dyspnea and fatigue Borg scores at the end of the walks. In healthy subjects, the primary outcome was the peak HR achieved during the walks. Walking speed and dyspnea and fatigue Borg scores at the end of the walks were included as secondary outcomes. All analyses were conducted using Stata 9.1 (StataCorp, College Station, TX, USA).

## Results

### Comparison of trails’ intensity—COPD patients

Patients were mostly male with normal BMI, and had mild (n = 1), moderate (n = 3), severe (n = 4) and very severe (n = 2) spirometric grades of COPD (Table 1). All of them completed the 6MWT and three intensity trails. One high intensity walk was excluded from the analysis due to technical problems with the gas analyzer sensor.

**Table 1:** Sociodemographic and clinical characteristics of COPD patients and healthy subjects.

		COPD patients n = 10	Healthy subjects n = 10
Men, n		9	5
Age (years), m (SD)		67 (9)	31 (4)
Weight (kg), m (SD)		68 (13)	66 (8)
Height (m), m (SD)		1.67 (0.09)	1.71 (0.09)
BMI (kg/m^2^), m (SD)		25 (4)	22 (2)
Comorbidities, n			
	Hypercholesterolemia	6	0
	Hypertension	4	0
	Chronic heart failure	3	0
	Diabetes	2	0
Current smoker, n		1	1
Dyspnea (mMRC grade, 0–4), n			
	1	5	0
	2	2	0
	3	3	0
FEV_1_ (% predicted), m (SD)		41 (18)	102 (11)
FEV_1_ (l), m (SD)		1.2 (0.5)	3.9 (0.6)
FVC (% predicted), m (SD)		67 (13)	103 (12)
FEV_1_/FVC (% ratio), m (SD)		44 (14)	81 (4)
TLC (% predicted), m (SD)		98 (18)	···
IC (% predicted), m (SD)		48 (19)	···
RV (% predicted), m (SD)		166 (55)	···
DLco (% predicted), m (SD)		51 (22)	···
DLco/VA (% predicted), m (SD)		63 (17)	…
6MWD (meters), m (SD)		448 (91)	…

BMI: Body Mass Index; mMRC: Modified Medical Research Council; FEV_1_: forced expiratory volume in one second post bronchodilator; FVC: forced vital capacity post bronchodilator; TLC: total lung capacity; IC: inspiratory capacity; RV: residual volume; DLco: diffusion capacity of the lung for carbon monoxide; DLco/VA: diffusion capacity of the lung for carbon monoxide to alveolar volume ratio; 6MWD: six min walking distance.

Table 2 shows that in COPD patients the peak values of $\dot{\text{V}}$O_2_, $\dot{\text{V}}$CO_2_, $\dot{\text{V}}$E, and HR increased significantly according to the trails’ intensity as also did energy expenditure (MET-min), duration of walking, walking speed and number of steps. However, the differences in physiological response between moderate and high intensity trails were not statistically significant (see S2 Table). The physiological response was higher in patients with severe-to-very severe COPD as compared to patients with mild-to-moderate COPD, but small sample size prevents against any formal statistical test. The physiological response to walking the low intensity trail was similar to that of the 6MWT, and walking the moderate or high intensity trails generated a statistically higher physiological demand (see S3 Table, comparing 6MWT with walking trails). On the low intensity trail, 9 patients maintained a walking speed of over 80% of the average speed achieved during the 6MWT; by contrast, on both moderate and high intensity trails, 7 patients achieved this.

**Table 2:** Physiological and clinical response to walks on three intensity trails in COPD patients.

	Low intensity trail	Moderate intensity trail	High intensity trail	*p*-trend
Peak $\dot{\text{V}}$O_2_ (mL/min/kg), m (SD)	15.9 (3.5)	17.4 (4.7)	17.7 (4.4)	0.02
Peak $\dot{\text{V}}$CO_2_ (mL/min/kg), m (SD)	13.3 (4.6)	14.7 (5.5)	15.1 (4.4)	0.02
Peak RER, m (SD)	1.0 (0.1)	1.1 (0.2)	1.0 (0.1)	0.48
Peak $\dot{\text{V}}$E (L/min), m (SD)	33.7 (5.1)	35.3 (6.7)	36.6 (7.2)	<0.01
Peak HR (beats/min), m (SD)	120 (18)	125 (20)	126 (20)	0.01
Energy expenditure volume (MET-min), m (SD)	60 (23)	64 (26)	72 (31)	<0.01
Walking time (s), m (SD)	994 (258)	1087 (337)	1136 (290)	<0.01
Walking speed (m/s), m (SD)	1.2 (0.3)	1.1 (0.3)	1.1 (0.3)	<0.01
Steps (n), m (SD)	1789 (536)	1856 (708)	2001 (576)	<0.01
Time for breaks (s), m (SD)	42 (101)	60 (91)	59 (112)	0.18
Final dyspnea (Borg score), median (p25-p75)	3 (2–4)	4 (3–5)	4 (3–5)	0.02
Final leg fatigue (Borg score), median (p25-p75)	2 (0–4)	2 (0–4)	2 (1–2)	0.21

$\dot{\text{V}}$O_2_: oxygen uptake; $\dot{\text{V}}$CO_2_: carbon dioxide production; RER: respiratory exchange ratio; $\dot{\text{V}}$E: min ventilation; HR: heart rate; MET: Metabolic Equivalent of Task.

### Comparison of spaces–Healthy subjects

The characteristics of young healthy subjects are shown in Table 1. Fifty percent were male and had normal BMI. All completed the 9 trails and no technical problems appeared. Table 3 shows no differences across trail spaces in the physiological and clinical response to walking trails of the same intensity. Peak HR increased steadily with trail intensity: mean (SD) 108 (10), 117 (10), and 123 (13) beats/min during low, moderate and high intensity trails, respectively (p-trend<0.001).

**Table 3:** Physiological and clinical response on identical intensity trails from three public spaces (boulevard, beach and park) in healthy subjects.

**Low intensity trail**	**Boulevard**	**Beach**	**Park**	***p*-value**
Peak HR (beats/min), m (SD)	110 (13)	107 (9)	108 (8)	0.74
Walking speed (m/s), m (SD)	1.5 (0.2)	1.7 (0.2)	1.6 (0.2)	0.03
Final dyspnea (Borg score), median (p25-p75)	0 (0–1)	0 (0–0)	0 (0–0)	0.40
Final leg fatigue (Borg score), median (p25-p75)	0 (0–2)	1 (0–2)	1 (0–2)	0.75
**Moderate intensity trail**	**Boulevard**	**Beach**	**Park**	***p*-value**
Peak HR (beats/min), m (SD)	120 (9)	114 (10)	116 (10)	0.40
Walking speed (m/s), m (SD)	1.5 (0.1)	1.7 (0.2)	1.5 (0.1)	0.02
Final dyspnea (Borg score), median (p25-p75)	0 (0–1)	0 (0–0)	0 (0–0)	0.36
Final leg fatigue (Borg score), median (p25-p75)	1 (0–1)	1 (1–2)	1 (1–2)	0.57
**High intensity trail**	**Boulevard**	**Beach**	**Park**	***p*-value**
Peak HR (beats/min), m (SD)	119 (12)	118 (10)	130 (15)	0.07
Walking speed (m/s), m (SD)	1.5 (0.1)	1.6 (0.2)	1.6 (0.2)	0.08
Final dyspnea (Borg score), median (p25-p75)	0 (0–1)	0 (0–1)	0 (0–1)	0.91
Final leg fatigue (Borg score), median (p25-p75)	1 (1–2)	2 (1–2)	2 (1–3)	0.71

HR: heart rate.

## Discussion

To our knowledge, this is the first study validating and testing the potential of self-paced walking trails designed in public spaces as a training method for COPD patients and it has produced two key findings. Firstly, both physiological response and energy expenditure in COPD patients increase with the trails’ intensity. Secondly, there were no observable differences in physiological response amongst the healthy control subjects to trails of the same intensity in the three different types of public spaces, validating the consistency of the methodology used for the trails’ design. These two findings deserve further interpretation.

First, the increase in $\dot{\text{V}}$O_2_, peak $\dot{\text{V}}$E and peak HR and MET-min according to the trails’ intensity was expected because by design we increased frequency, length and/or slope of urban elements (stairs, ramps and types of surfacing) which overall augment the trails’ intensity. It is interesting to note that the trail’s different contents (in terms of urban elements) could lead to different patterns of physiological response. For example, in our case study for COPD patients (the boulevard trail) there were small differences in physiological responses between moderate and high intensity trails which were not statistically significant. This could be attributed to several factors, such as the small sample size combined with small effect difference and large variability of outcomes, or the use of absolute parameters of intensity instead of relative to the maximal capacity of each patient. These factors should be acknowledged as limitations of the current research. Also, the fact that patients were instructed to keep a self-regulated pace could have made them adapting to the high intensity trail by reducing speed, increasing resting time or simply not finishing the walk. This suggests that the future use of such urban trails for the training of COPD patients needs to be accompanied with clear instructions of keeping a dyspnea Borg scale between 4 and 6, as recommended in traditional rehabilitation programs [11]. Finally, it could be argued that the frequency of stairs and ramps was increased between the moderate and high intensity trail but without increasing their length or height (because the space did not allow it). By contrast, the presence of sand on the beach trail resulted in higher HR peak to be reached, as higher stairs did on the park trail. This would support that the volume of energy expenditure reflects better the level of the trails’ physiological demand than the peak values of physiological response.

Our second observation relates to the physiological response while walking on each intensity trail in young healthy subjects that did not vary across space types (boulevard, beach and park). Our study highlights that urban elements, such as stairs, ramps and sand, can be standardized in terms of height, length and slope, thus allowing their combination to be used for the design of trails in other geographical areas. How they are combined in terms of increased frequency or increased length will depend on the possibilities of each public space.

Until now, research has been scarce in relation to the physiological demands that these urban elements may present to in COPD patients when left to walk at their own-pace. Castro *et al*. [26] showed that, in mild-to-very severe COPD patients, walking up or down 13 m of a ramp at 10% inclination or 17 steps of stairs produced a 2 to 3-fold increase in $\dot{\text{V}}$O_2_ compared to resting conditions. Similarly, Jeng *et al*. [28] found that walking on level ground and walking upstairs required higher $\dot{\text{V}}$O_2_ than sitting or standing, although in this study the walking speed was set by design. Barberan-Garcia *et al*. [6] showed in COPD patients that a 6-min Nordic Walking test performed on sand generates significantly higher $\dot{\text{V}}$O_2_, $\dot{\text{V}}$E, HR, dyspnea and leg fatigue values than the same test on firm ground. Along with these previous data, our findings suggest that it is feasible to design trails for training COPD patients using stairs, ramps and sand by capitalizing on the high energy expenditure that walking in these conditions demands from these patients.

Several arguments suggest that physiological responses in COPD patients to walking trails can be interpreted in terms of response to typical exercise training interventions. First, the patients in the current study walked mostly at a speed of above 80% of the average speed achieved during 6MWT, which has been proposed as a target for walking training [5,29]. Secondly, our patients achieved a mean of 4 points on the Borg dyspnea scale after all trails, in keeping with the 4 to 6 score often considered a target for training intensity [11]. Altogether our findings suggest that walking on the Urban Training^TM^ trails, when appropriately designed, could be used as an effective training intervention for COPD patients customizing individually the duration, frequency and intensity of the interventions, thus embracing the large variability in individual goals and possibilities.

From a public health viewpoint, our approach offers the possibility of training to a vast majority of COPD patients irrespective of their individual characteristics. This is specifically relevant for the mild-to-moderate COPD patients due to two identified factors: (1) they do not usually have access to the conventional tertiary-based training interventions because the latter are prescribed usually in advanced stages of the disease; and (2) they cannot benefit from public policies targeted at the general population because the resulting walking routes are not adapted to the physiological and functional limitations of the disease population, principally because of their excessive length (mean (SD) 3.4 (0.7) km) [30,31], or steep slopes and stairs.

Strengths of our study were the standardization of the methodology used to design the trails, which included visiting the spaces, measuring urban elements, scoring and categorizing their intensity and finally combining them. Similarly, we measured physiological response directly by a device that provides several outcomes of energy expenditure that have been previously used in COPD patients[6]. We carefully standardized data collection by randomizing the order of the trails’ intensity in the different public spaces, and taking into account atmospheric conditions and patients’ clinical status. The current study has also limitations. First, the lack of a maximal exercise test precluded the use of relative values of exercise intensity that could have helped in the interpretation of the data. However, the burden on COPD patients was already too high because of the need for four days for different walking-related activities, and the 6MWT is accepted as a standardized and reliable tool that provides valuable information in terms of exercise capacity. Second, the external validity of the study may be challenged because both patients and healthy subjects were volunteers, but they were not selected on the basis of their physical activity or exercise capacity levels. Finally, the exclusion of patients on long-term oxygen therapy (because of its incompatibility with the gas analyzer) prevents extrapolating the current results to the most severe subset of COPD patients.

In conclusion, we validated the trails design for the training of COPD patients by showing that the physiological response and the energy expenditure on unsupervised walking on these trails increased according to the predefined trails’ intensity and did not change across trails of the same intensity and different public space. Interventions combining walking these trails with other strategies, such as behavioral change interventions, may be a feasible and effective way to promote physical activity for COPD patients in the community.

## Supporting Information

S1 Image Reproduction of the Urban Training^TM^ trails (low, moderate and high intensity) from a boulevard space (Platja de la Barceloneta), a beach space (Platja de Nova Icària) and a park space (Parc de la Ciutadella). (DOCX)

S2 Image A COPD patient walking a high intensity trail in a boulevard space. (DOCX)

S1 Table Study trails’ characteristics (three intensity trails of each three public spaces). (DOCX)

S2 Table Comparison of physiological and clinical response to walks on moderate and high intensity trails in COPD patients. (DOCX)

S3 Table Comparison of physiological response in 6-min walking test and walking three different intensity trails in COPD patients. (DOCX)

S1 Text Design of trails–length, content and depiction of the trails. (DOCX)
