# Supplementary material for: Validation of Walking Trails for the Urban TrainingTM of Chronic Obstructive Pulmonary Disease Patients
Source: PLoS One. 2016 Jan 14;11(1):e0146705. doi: 10.1371/journal.pone.0146705 (PMC4713200; doi:10.1371/journal.pone.0146705)
Supplement: S1 Image — (DOCX) [file pone.0146705.s001.docx]

**S1 Image. Reproduction of the Urban Training^TM^ trails (low, moderate and high intensity) from a boulevard space (Platja de la Barceloneta), a beach space (Platja de Nova Icària) and a park space (Parc de la Ciutadella).**

** Boulevard**

* Note that this trail is close to a beach space but belongs to a boulevard space because walking happens on the sidewalks of the thoroughfare. Consequently, sand is not used in this case as an intensity element.

**Beach**

**Park**
