# Supplementary material for: Validation of Walking Trails for the Urban TrainingTM of Chronic Obstructive Pulmonary Disease Patients
Source: PLoS One. 2016 Jan 14;11(1):e0146705. doi: 10.1371/journal.pone.0146705 (PMC4713200; doi:10.1371/journal.pone.0146705)
Supplement: S2 Image — (DOCX) [file pone.0146705.s002.docx]

**S2 Image. A COPD patient walking a high intensity trail in a boulevard space.**


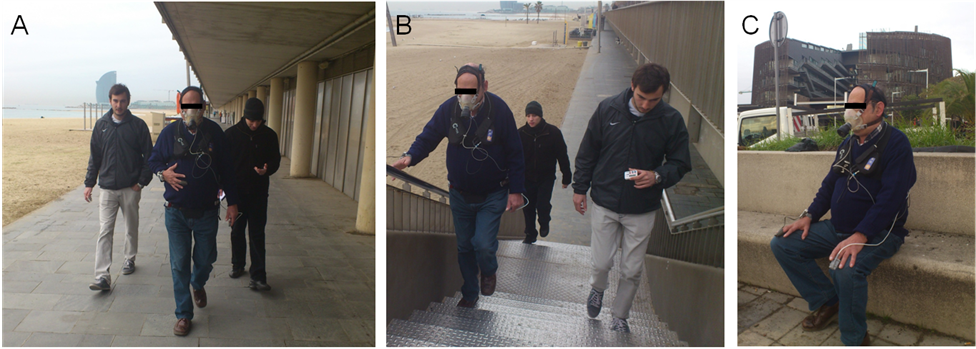
Male COPD patient wearing the MetaMax® 3B device while walking a high intensity trail. A: Walking on level ground. B: Stair climbing. C: Resting after the trail.
