# Supplementary material for: Validation of Walking Trails for the Urban TrainingTM of Chronic Obstructive Pulmonary Disease Patients
Source: PLoS One. 2016 Jan 14;11(1):e0146705. doi: 10.1371/journal.pone.0146705 (PMC4713200; doi:10.1371/journal.pone.0146705)
Supplement: S1 Table — (DOCX) [file pone.0146705.s003.docx]

**S1 Table. Study trails’ characteristics (three intensity trails of each three public spaces).**

|  | **Boulevard** | | | |  | **Beach** | | |  | **Park** | | | | |
| --- | --- | --- | --- | --- | --- | --- | --- | --- | --- | --- | --- | --- | --- | --- |
| Intensity | Low | Moderate | High | |  | Low | Moderate | High |  | Low | Moderate | | High | |
| Distance, m | 1340 | 1400 | 1430 | |  | 1009 | 1006 | 1160 |  | 1270 | 1670 | | 1690 | |
| Up-Stair  *Steps*  *Slope, %*  *Height, m* | 0 | 2  23  25  3.5 | 2  23  25  3.5 | |  | 0 | 0 | 1  14  42  2 |  | 0 | 3 | | 2 | |
|  |  |  |  |  |  |  |  |  |  |  | 10  30  1.5 | 26  28  3 | 10  30  1.5 | 50  28  9 |
| Down-stair  *Steps, n*  *Slope, %*  *Height, m* | 0 | 2  23  25  3.5 | 1  23  25  3.5 | |  | 0 | 0 | 0 |  | 0 | 2  26  28  3 | | 1  50  28  9 | |
| Up-hill  *Slope, %*  *Height, m* | 0 | 0 | 1  3.5  3.5 | |  | 0 | 3  8  2.5 | 1  8  2.5 |  | 0 | 0 | | 1  11  3 | |
| Down-hill  *Slope, %*  *Height, m* | 0 | 0 | 2 | |  | 0 | 3  8  2.5 | 2  8  2.5 |  | 0 | 0 | | 1  15  3 | |
|  |  |  | 9  3.5 | 5  3.5 |  |  |  |  |  |  |  |  |  |  |
| Sand stretch  *Distance, m* |  |  |  | |  | 0 | 0 | 1  100 |  |  |  | |  | |
| Intensity elements, n | 0 | 4 | 6 | |  | 0 | 6 | 5 |  | 0 | 5 | | 5 | |
