# Supplementary material for: Validation of Walking Trails for the Urban TrainingTM of Chronic Obstructive Pulmonary Disease Patients
Source: PLoS One. 2016 Jan 14;11(1):e0146705. doi: 10.1371/journal.pone.0146705 (PMC4713200; doi:10.1371/journal.pone.0146705)
Supplement: S2 Table — (DOCX) [file pone.0146705.s004.docx]

**S2 Table. Comparison of physiological and clinical response to walks on moderate and high intensity trails in COPD patients.**

|  | **Moderate**  **intensity trail** | **High**  **intensity trail** | ***p*-value** |
| --- | --- | --- | --- |
| Peak V̇O_2_ (mL/min/kg), m (SD) | 17.4 (4.7) | 17.7 (4.4) | 0.90 |
| Peak V̇CO_2_ (mL/min/kg), m (SD) | 14.7 (5.5) | 15.1 (4.4) | 0.83 |
| Peak RER, m (SD) | 1.1 (0.2) | 1.0 (0.1) | 0.52 |
| Peak V̇E (L/min), m (SD) | 35.3 (6.7) | 36.6 (7.2) | 0.67 |
| Peak HR (beats/min), m (SD) | 125 (20) | 126 (20) | 0.89 |
| Energy expenditure volume (MET-min), m (SD) | 64 (26) | 72 (31) | 0.51 |
| Walking time (s), m (SD) | 1087 (337) | 1136 (290) | 0.73 |
| Walking speed (m/s), m (SD) | 1.1 (0.3) | 1.1 (0.3) | 0.74 |
| Steps (n), m (SD) | 1856 (708) | 2001 (576) | 0.62 |
| Time for breaks (s), m (SD) | 60 (91) | 59 (112) | 0.99 |
| Final dyspnea (Borg score), median (p25-p75) | 4.5 (3-5) | 4 (3-5) | 0.73 |
| Final leg fatigue (Borg score), median (p25-p75) | 2 (0-4) | 1.5 (1-2) | 0.76 |

V̇O_2_: oxygen uptake; RER: respiratory exchange ratio; V̇CO_2_: carbon dioxide production V̇E: minute ventilation; HR: heart rate. MET: Metabolic Equivalent of Task.
