# Supplementary material for: Validation of Walking Trails for the Urban TrainingTM of Chronic Obstructive Pulmonary Disease Patients
Source: PLoS One. 2016 Jan 14;11(1):e0146705. doi: 10.1371/journal.pone.0146705 (PMC4713200; doi:10.1371/journal.pone.0146705)
Supplement: S3 Table — (DOCX) [file pone.0146705.s005.docx]

**S3 Table. Comparison of physiological response in 6-min walking test and walking three different intensity trails in COPD patients.**

| **n=10** | **6 MWT** | **Low** | ***p*-value^*^** | **Moderate** | ***p-*value^**^** | **High** | ***p*-value^***^** |
| --- | --- | --- | --- | --- | --- | --- | --- |
| Peak V̇O_2_ (mL/min/kg), m (SD) | 15.5 (3.8) | 15.9 (3.5) | 0.34 | 17.4 (4.7) | 0.12 | 17.7 (4.4) | 0.05 |
| Peak V̇CO_2_ (mL/min/kg), m (SD) | 13.8 (2.5) | 13.3 (4.6) | 0.54 | 14.6 (5.5) | 0.46 | 15.1 (4.4) | 0.21 |
| Peak RER (V̇CO_2_ / V̇O_2_), m (SD) | 1.0 (0.1) | 1.0 (0.1) | 0.46 | 1.1 (0.2) | 0.15 | 1.0 (0.1) | 0.23 |
| Peak V̇E (L/min), m (SD) | 32.4 (5.4) | 33. 7 (5.1) | 0.25 | 35.3 (6.7) | 0.05 | 36.6 (7.2) | 0.02 |
| Peak HR (beats/min), m (SD) | 117 (17) | 120 (18) | 0.31 | 125 (20) | <0.01 | 126 (20) | 0.01 |
| Time for breaks (s), m (SD) | 14 (29) | 42 (101) | 0.30 | 60 (91) | 0.05 | 59 (112) | 0.13 |
| Mean walking speed (m/s), m (SD) | 1.3 (0.2) | 1.2 (0.3) | 0.04 | 1.1 (0.3) | <0.01 | 1.1 (0.3) | <0.01 |

6MWT: 6 min walking test; V̇O_2_: oxygen uptake; RER: respiratory exchange ratio; V̇CO_2_: carbon dioxide production V̇E: minute ventilation; HR: heart rate.

^*^p-value 6MWT *vs* Low intensity trail

^**^ p-value 6MWT *vs* Moderate intensity trail

^***^ p-value 6MWT *vs* High intensity trail
