# Supplementary material for: Validation of Walking Trails for the Urban TrainingTM of Chronic Obstructive Pulmonary Disease Patients
Source: PLoS One. 2016 Jan 14;11(1):e0146705. doi: 10.1371/journal.pone.0146705 (PMC4713200; doi:10.1371/journal.pone.0146705)
Supplement: S1 Text — (DOCX) [file pone.0146705.s006.docx]

**S1 Text. Design of trails – length, content and depiction of the trails.**

Low intensity trails were flat and 720 to 1,700 m length without intensity components. Moderate intensity trails were built based on the low intensity trails but adding some intensity elements (2 to 5 stairs or ramps, depending on their intensity score), and/or increasing the length (up to 2,120 m). High intensity trails were the longest (up to 2,440 m) and had more intensity elements (3 to 8 stairs or ramps) or a higher frequency of the same elements than the moderate intensity trails. Each trail was drawn as a map on A4 piece of paper in a 1:2.500 scale. Prior to the study, we pilot tested these maps in 12 COPD patients and their family members from Hospital del Mar, and in 20 elderly subjects passers-by the spaces. They reviewed different versions of the drawings to check for a correct understanding and interpretation of the information reflected on the maps. S1 Image shows the three intensity trails of the three public spaces (boulevard, beach and park) used in this study. The physical characteristics of the nine study trails are detailed in S1 Table.
